# Trametinib: Could It Be a Promising Drug to Treat Atypical Chronic Myeloid Leukemia?

**DOI:** 10.7759/cureus.26619

**Published:** 2022-07-06

**Authors:** Marwa Elsayed, Stephanie Harry, Suprana Nanua, Shayaan Zaidi, Muhammad H Habib, Shahzad Raza

**Affiliations:** 1 Internal Medicine, University of Missouri Kansas City School of Medicine, Kansas City, USA; 2 Hematology/Oncology, Saint Francis Cancer Center Warren Clinic, Tulsa, USA; 3 Oncology, Saint Luke's Cancer Institute, University of Missouri Kansas City, Kansas City, USA; 4 Oncology, University of Kansas School of Medicine, Kansas City, USA; 5 Oncology, Rutgers Cancer Institute of New Jersey, New Brunswick, USA

**Keywords:** atypical chronic myeloid leukemia, chronic myeloid leukemia, myelodysplastic (mds)/myeloproliferative neoplasm (mpn) disease spectrum, myelodysplastic/myeloproliferative diseases, trametinib, next-generation sequencing studies, atypical chronic myelogenous leukemia

## Abstract

Atypical chronic myeloid leukemia (aCML) is a rare disease that is currently classified under the myelodysplastic (MDS)/myeloproliferative neoplasm (MPN) disease spectrum. MDS/MPN diseases are characterized by the absence of the Philadelphia (Ph) chromosome and the overlap between bone marrow fibrosis and dysplastic features. The Ph chromosome, resulting from BCR-ABL1 translocation, helps to distinguish aCML from chronic myeloid leukemia (CML). The currently reported incidence of aCML is imprecise because aCML is diagnosed primarily based on morphological features and other unspecified laboratory findings, and there is an especially high chance of under-diagnosis of aCML and other MDS/MPN diseases. Recent advances in next-generation sequencing (NGS) have allowed a greater understanding of the nature of aCML, providing better opportunities to achieve higher diagnostic accuracy and for the use of more targeted treatment to achieve better outcomes. Herein, we present a case of a 68-year-old woman who came to our hospital complaining of shortness of breath, fatigue, and weakness, who was found to have significantly increased leukocytosis, hepatosplenomegaly, and was negative for the Ph chromosome. Further investigations with NGS revealed mutations in ASXL1, GATA2, NRAS, and SRSF2 but not CSF3R. In addition to this, peripheral smear and bone marrow aspiration findings were suggestive of aCML based on specific morphological findings. Since the patient was ineligible for a stem cell transplant (SCT), symptomatic treatment was started with cell transfusion; however, the patient continued to have symptomatic anemia that required multiple transfusions. A trial with trametinib, a mitogen-activated protein kinase kinase (MEK) inhibitor, was later started as a targeted therapy based on one of her genetic mutations. Interestingly, the patient’s blood counts stabilized, she reported feeling better, and she did not need any blood transfusions for four consecutive months during treatment with trametinib. Unfortunately, our patient later died from sepsis resulting from secondary infections. In light of the significant advancements in NGS, clinicians should always consider utilizing it as a helpful tool to not only establish a rare diagnosis of aCML but also to offer the best available targeted therapy when applicable. This might alleviate the burden associated with the poor prognosis of aCML.

## Introduction

Chronic myeloid leukemia (CML) that is negative for the Philadelphia (Ph) chromosome has historically been considered a subtype of CML with a poor prognosis. In 2016, the World Health Organization released a new classification of myelodysplastic (MDS)/myeloproliferative neoplasm (MPN) diseases that are negative for the Ph chromosome, including atypical chronic myeloid leukemia (aCML), chronic myelomonocytic leukemia (CMML), chronic neutrophilic leukemia (CNL), and MDS/MPN-unclassified (MDS/MPS-U). The reported incidence of aCML is as low as one to two per 100 cases of confirmed CML, which are positive for the Ph chromosome [1,2]. aCML peaks during the seventh and eighth decades, and predominantly affects males. Because of its rarity, studies have shown significant variations in the overall survival (OS) rate for aCML, with an average OS of between 14 and 30 months [3]. The most common factors associated with poor prognosis of aCML include female gender, age of more than 65 years, elevated WBC > 50 x 109/L, and increased precursors in peripheral blood [2,4,5]. Recent data have revealed that a low hemoglobin level < 10 g/dL and certain mutations, particularly in TET2 and ETNK1, are associated with a more adverse outcome [6].

## Case presentation

Our patient was a 68-year-old female with multiple comorbidities, including but not limited to chronic obstructive pulmonary disease (COPD), hypothyroidism, asthma, diabetes, osteoarthritis, peptic ulcer disease, hypertension, obstructive sleep apnea, and previous history of grade 2 endometrial adenocarcinoma status following total abdominal hysterectomy, bilateral salpingo-oophorectomy, and radiation in 2008. She presented to our hospital with shortness of breath and reported feeling weak and fatigued, having dyspnea upon exertion, palpitations, chest heaviness upon exertion, night sweats, hot flashes, and unintentional weight loss of 23 lb over the past three months prior to presentation. The physical examination was positive for splenomegaly. The initial complete blood count (CBC) was significantly positive for leukocytosis with a total white blood cell (WBC) count of 495.36 TH/µL, hemoglobin of 9.1 g/dL, and a platelet count (PLT) of 95 TH/µL. Subsequent manual differential revealed 48% neutrophils, 2% blasts, 0% eosinophils, 0% basophils, 0% monocytes, 31% myelocytes, 11% metamyelocytes, 7% promyelocytes, and 1% lymphocytes. Biochemical analysis was positive for elevated uric acid and lactate dehydrogenase at 15.4 mg/dL and 4882 IU/L, respectively (Table 1). BCR-ABL flow cytometry results were negative for the Ph chromosome.

**Table 1:** Remarkable blood test results during the treatment course

	1st presentation	During treatment course	After 6 cycles of trametinib
WBC (TH/µL)	495.36	36.37	68.87
Hemoglobin (g/dL)	9.1	4.7	8.2
Platelets (TH/µL)	95	60	200
Blast %	2	12	4
Neutrophils %	48	51	51
Lymphocytes %	1	3	5
Monocytes %	0	1	0
Eosinophils %	0	0	0
Basophils %	0	1	1
Myelocytes %	31	15	29
Metamyelocytes %	11	14	10
Promyelocytes %	7	3	3
Uric acid (mg/dL)	15.4	6.1	6.8
Lactate dehydrogenase (IU/L)	4,882	1,671	2,541

**Figure 1:**
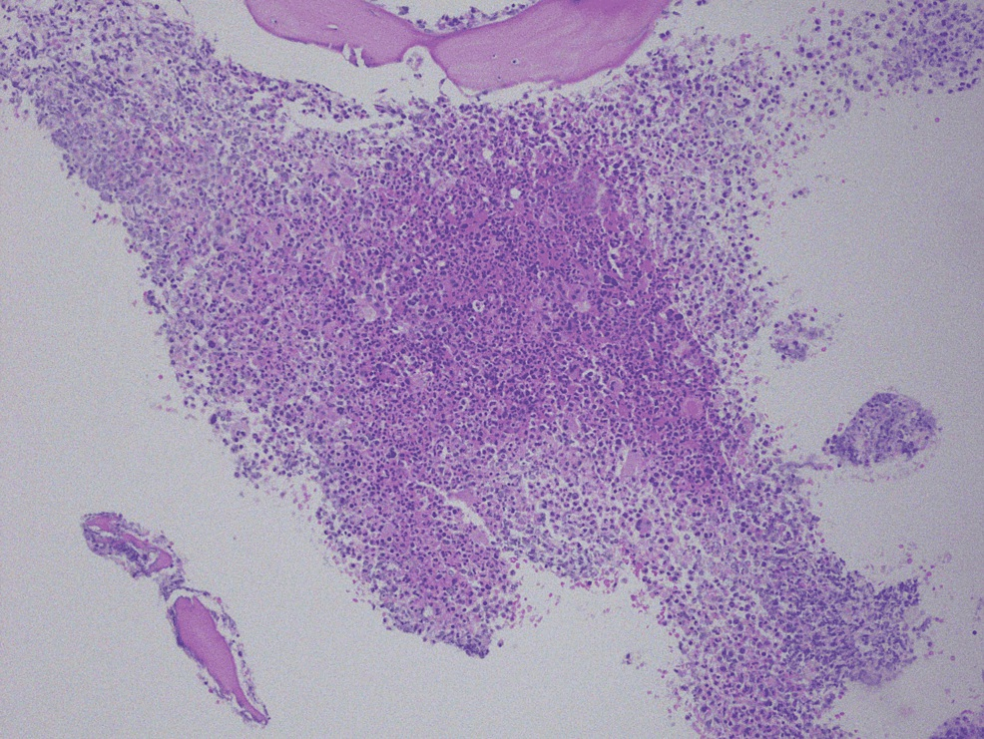
Hypercellularity with myeloid hyperplasia and megakaryocyte atypia at 100x power

**Figure 2:**
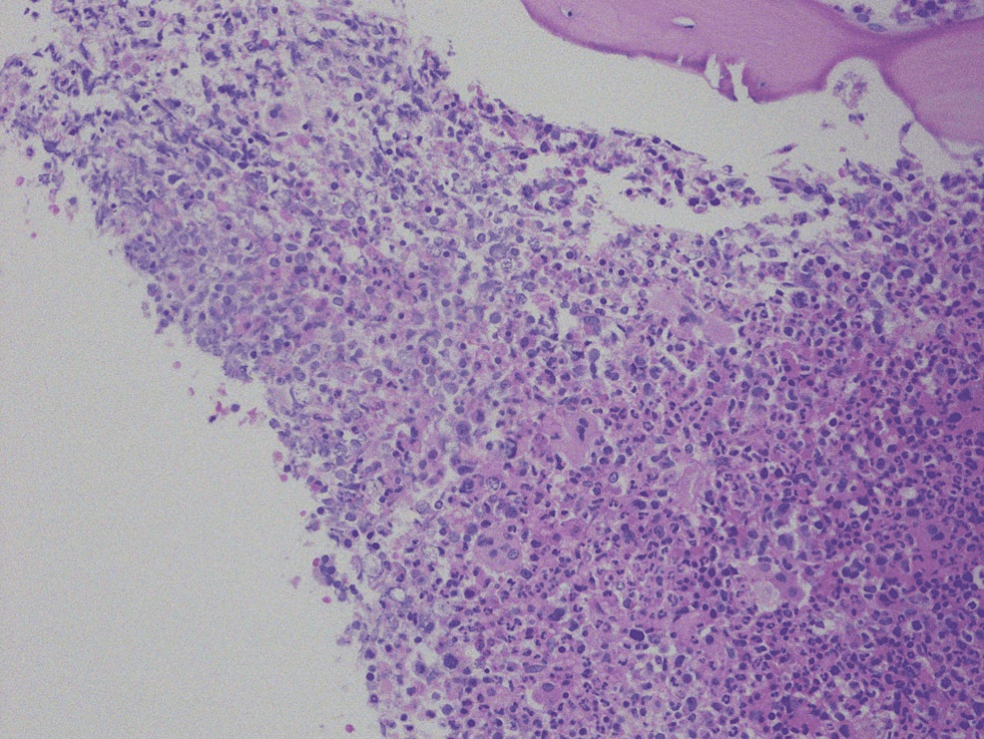
Hypercellularity with myeloid hyperplasia and megakaryocyte atypia at 200x power

**Figure 3:**
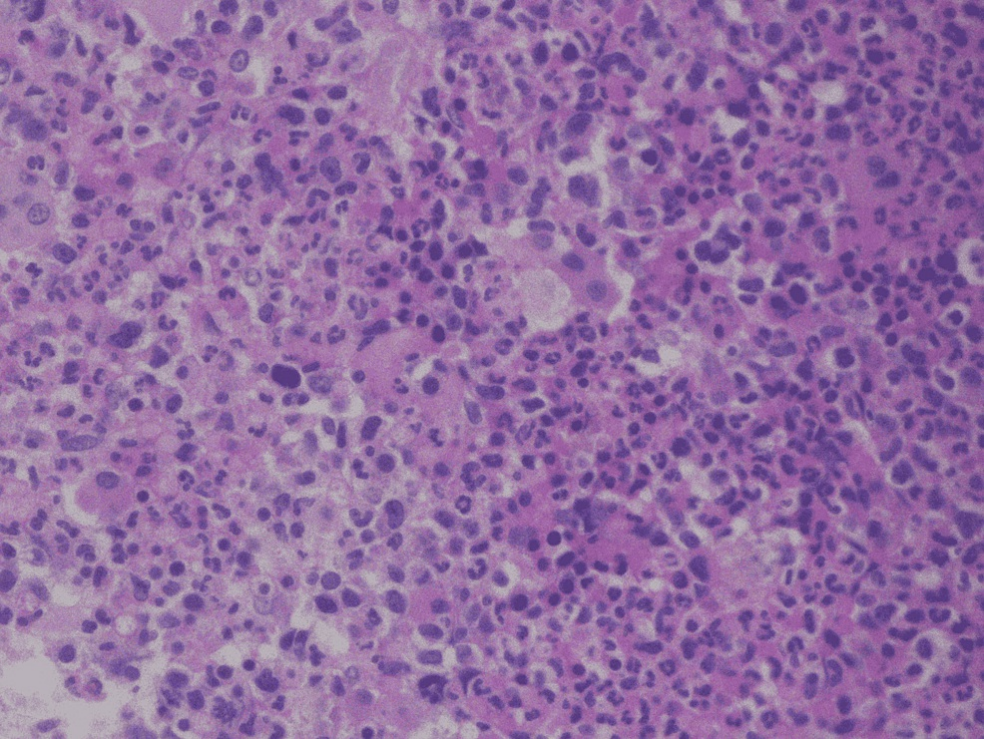
Hypercellularity with myeloid hyperplasia and megakaryocyte atypia at 400x power

The patient’s previous history of radiation exposure increased her risk of MDS/MPN diseases. A bone marrow biopsy with aspiration analysis was significant for hypercellular marrow with dysmyelopoiesis, and no blasts were seen (Figures 1-3).

Next-generation sequencing (NGS) revealed mutations in ASXL1, GATA2, NRAS, and SRSF2 without CSF3R or SETBP1 mutations. The patient was sent for evaluation by a local bone marrow transplant center. She was deemed not a good candidate for an allogeneic stem cell transplant due to a lack of social support and access to a 24-hour caregiver.

The patient was started on hydroxyurea for the management of her symptoms; however, even with a subsequent increase in the dose of hydroxyurea that reached 1.5 g twice daily, her WBC count remained challenging to manage. Therefore, treatment with a hypomethylating agent with decitabine at 20 mg/m^2^, Monday to Friday, was initiated in addition to continuation with hydroxyurea. Despite receiving a total of four cycles of decitabine in addition to hydroxyurea, she continued to have persistent anemia requiring blood transfusions. Repeated bone marrow biopsy showed dysmyelopoiesis and fibrosis with 13% blasts and, therefore, venetoclax was added to the regimen. The patient remained on venetoclax, hydroxyurea, and decitabine for approximately four months. The decision was made to discontinue venetoclax and hydroxyurea due to the patient’s worsening anemia thrombocytopenia, increasing blasts, and development of cardiac toxicity from venetoclax in the form of pericardial effusion and non-ischemic cardiomyopathy. Following six cycles of decitabine, and considering she harbored NRAS mutation based on the NGS result, she was ultimately started on a mitogen-activated protein kinase kinase (MEK) inhibitor, trametinib (2 mg), once daily only. Her blood counts subsequently stabilized, and she began to feel better and less fatigued. She did not require any blood transfusions for four months while on this therapy.

Unfortunately, she was later hospitalized for approximately three weeks for sepsis secondary to pneumonia, encephalopathy, frequent falls, and a *Clostridium difficile* infection. During this period of hospitalization, she required a blood transfusion and did not receive trametinib. Due to her debility and weakness, she was discharged to a nursing home. Shortly after that, she was admitted for injury secondary to falls, hypotension, and sepsis secondary to large decubitus ulcers. The patient had stopped taking trametinib since her previous hospitalization. Her WBC count was 108,000 TH/µL, and peripheral blasts had increased by 20%. The peripheral blood smear was significantly positive for transformation to acute leukemia with approximately 29% blasts. At this time, due to her considerable deconditioning and multiple medical comorbidities, the decision was made to not pursue cancer-directed treatment and to proceed with comfort care. Unfortunately, the patient died from septic shock secondary to an infected bed sore.

## Discussion

Clinical presentation and diagnostic criteria

The most common presenting clinical scenario for aCML is related to the associated bone marrow (BM) depletion symptoms, including but not limited to anemia (e.g., fatigue and dyspnea), thrombocytopenia (e.g., bleeding tendency), and BM-associated fibrosis (e.g., hepatosplenomegaly) [7,8]. The initial diagnostics workup must include CBC with manual differential, complete metabolic panel (CMP), and BM biopsy and aspiration. Similar to CML, aCML is characterized by the presence of myeloid lineage hyperplasia of the BM in addition to peripheral leukocytosis, with evidence of premature myeloid precursors in the peripheral smear (PS) [9]. In 2001, the World Health Organization (WHO) issued a new classification for MDS/MPN overlap syndrome characterized by both MDS and myeloproliferative features at the time of diagnosis [10]. This new classification comprises a spectrum of different myelogenous malignancies, including aCML, chronic myelomonocytic leukemia (CMML), and chronic neutrophilic leukemia (CNL). In 2016, the WHO released a new criterion to distinguish aCML from CML and other MDS/MPN overlap syndromes, as shown in Table 2 [11].

**Table 2:** Diagnostic criteria for aCML (BCR-ABL1) aCML: atypical chronic myeloid leukemia; PB: peripheral blood; BM: bone marrow; CML: chronic myeloid leukemia; PMF: primary myelofibrosis; PV: polycythemia vera; ET: essential thrombocythemia.

aCML diagnostic criteria
• PB leukocytosis due to increased numbers of neutrophils and their precursors (promyelocytes, myelocytes, metamyelocytes) comprising ≥10% of leukocytes
• Dysgranulopoiesis, which may include abnormal chromatin clumping
• No or minimal absolute basophilia; basophils usually <2% of leukocytes
• No or minimal absolute monocytosis; monocytes <10% of leukocytes
• Hypercellular BM with granulocytic proliferation and granulocytic dysplasia, with or without dysplasia in the erythroid and megakaryocytic lineages
• <20% blasts in the blood and BM
• No evidence of *PDGFRA*, *PDGFRB*, *FGFR1* rearrangement, or *PCM1*-*JAK2*
• Not meeting WHO criteria for *BCR-ABL1*^+^ CML, PMF, PV, or ET

aCML is typically characterized by leukocytosis with a WBC count of more than 13 TH/µL, mainly neutrophilic with a left shift (at least 10% immature granulocytes with severe dysplastic granulopoiesis features, e.g., hypogranular and hypolobated neutrophils, abnormal chromatin clumping, and pseudo-Pelger-Hüet neutrophils), less countable monocytosis (<10%), and basophilia (<2%) in the peripheral blood. Typically, aCML is associated with <20% blasts in the PS [1,12,13]. BM aspirate features in aCML are characterized by hypercellularity with significant dysplastic granulocytes, frequently dysplastic erythroid precursors, and, to a lesser extent, megakaryocyte progenitors [14,15]. These certain features are important for distinguishing aCML from other myelogenous diseases, e.g., aCML is associated with less basophilia and more dysplastic features compared to CML, as well as less monocytosis compared to CMML.

Molecular pathogenesis

Though the landscape of the molecular pathogenesis of MDS/MPN is yet to be clearly defined, the acute surge in NGS usage has led to a better understanding of MDS/MPN. There is no single mutation that can define a singular MDS/MPN disease, but certain mutations can be found at a higher percentage in certain types of MDS/MPN diseases, which can make diagnosis more feasible, especially if the morphological findings are vague. Moreover, certain mutations can guide tailored targeted therapy and serve as a prognostic factor. Genetic mutations in SETBP1, ASXL1, N/KRAS, SRSF2, and TET2 can be identified in more than 20% of aCML cases [4,16,17].

SETBP1 is considered a high-risk mutation of aCML. A retrospective study by Wang et al. investigated 134 patients diagnosed with either aCML or MDS/MPN-U disorders from seven large medical centers to evaluate which features may be characteristic for both groups. Interestingly, SETBP1 mutations were detected with a higher incidence in the subgroup of patients with highly elevated WBCs and with lower hemoglobin and platelet counts, a prognostic factor that is usually associated with poor prognosis [4]. Similar findings were documented in a retrospective study by Meggendorfer et al. that analyzed genetic mutations in 1130 patients diagnosed with either MPN or MDS/MPN [18]. In addition, a strong correlation was revealed between SETBP1 and ASXL1 based on the analysis obtained in that study. A lower survival rate for aCML patients with SETBP1 mutations was also described in the works of Piazza et al. and Damm et al. [16,19]. NRAS mutations are another type of mutation reported in 35% of aCML patients in one study [4]. N/KRAS mutations are another type of mutation reported in more than 20% of aCML patients, as mentioned above. NRAS mutations are one of the core mutations that play a major role in over-activating the mitogen-activated protein kinase (MAPK) pathway, which contributes to unregulated leukemogenesis in leukemoid malignancies [20]. Targeting this overactivated MAPK pathway with inhibitors is one of the new strategies to treat aCML patients with mutated N/KRAS that is currently under investigation in clinical trials. CSF3R and JAK2 V617F are two other targetable mutations reported in aCML; however, their incidences are very low [21].

Treatment

To date, no defined algorithm or risk-stratified approach for aCML has been established. Because of the aggressive nature of aCML, the wait-and-watch approach typically cannot be applied in most cases. Hematopoietic stem cell transplantation (HSCT) has been reported in sporadic studies with inconsistent outcomes. In a retrospective study, Mittal et al. investigated a total of 20 patients (five diagnosed with myelofibrosis, eight diagnosed with CMML, and seven diagnosed with aCML) who underwent HSCT between 1991 and 2001 [22]. Five patients had died by 3-26 months after HSCT, secondary to either graft versus host disease (GVHD), disease progression, sepsis, or unknown causes; however, this result was not reliable due to the heterogenicity of induction therapy and the inconsistency in related versus unrelated donors. By contrast, Koldehoff et al. presented a favorable outcome for nine patients with aCML who were treated with HSCT [23]; eight patients remained alive at the median follow-up of 55 months (range: 9-118 months). Interestingly, all patients remained in remission after HSCT; one patient underwent his second peripheral HSCT because of a relapse, with more than 20% lymphocytic blasts in the BM and peripheral blood after 19 months from his first synergistic HSCT. It is important to mention that this high rate of median overall survival was at the expense of an increasing rate of acute GVHD (62.5%), with chronic GVHD occurring in most of the eight cases that were treated with allogeneic transplantation. On a side note, HSCT therapy is highly recommended for patients with poor prognostic factors, e.g., significant anemia, a high burden of blasts, and mutations associated with increased mortality, such as SETBP1 and ASXL1 [21,24].

There are no large control trials that have established the effectiveness of cytoreductive agents in treating aCML; however, based on their role in treating other myelogenous malignancies, they are usually recommended either as a bridge therapy until initiating the HSCT process or as palliative therapy for patients who are not eligible for HSCT [25]. Different results have been reported for different agents. Hydroxyurea (HU) and interferon-α (IFN-α) have been widely used in BCR-ABL-negative MPN to control abundant leukocytosis, circulating blasts, and splenomegaly with complete or partial hematological responses but show a short response duration [26,27]. Recently, pegylated interferon-α-2β was proven to be safer with regard to toxicity and tolerability outcome (once-weekly dose) compared to the regular IFN-α [14,28]. Hypomethylating agents (HMAs), particularly azacytidine or decitabine, have recently and sporadically been used in symptomatic treatment for aCML. Decitabine achieved superior results compared to azacytidine in terms of achieving a hematological response but did not improve the reported median OS rates for aCML [29-34].

Targeted therapy has frequently been introduced in clinical practice of late. Though there is no defining genetic mutation for aCML yet, some targetable mutations can be used to tailor individual therapy plans. The main targetable mutations in aCML include NRAS, SETBP1, the rarely reported JAK2 VS17F, and CSF3R [35].

Ruxolitinib has been successfully used in treating myelofibrosis and polycythemia vera patients harboring JAK2 VS17F mutations that are resistant to HU therapy [36,37]. Based on this, Assi et al. evaluated the role of ruxolitinib in a phase II clinical trial based on MDS/MPN diseases, either with or without azacytidine, and demonstrated a clinical response in patients, particularly in patients with mutated JAK2 VS17F [38]. It is important to mention that the rate of response was low in aCML cases compared to MDS/MPN-U, but it is not clear if this is due to a less significant number of aCML cases (four out of a total of 35) or because of other mutations present in aCML cases that might affect the response to ruxolitinib, e.g., SETBP1.

Dasatinib, an SRC tyrosine kinase inhibitor, is another targeted therapy that targets CSF3R mutations, particularly CSF3R truncation mutations, based on in vitro trials [39]. Though dasatinib has not been used in large clinical trials, including MDS/MPN diseases that are associated with CSF3R truncated mutations, it has shown a favorable outcome when used in a patient with B-cell acute lymphoblastic leukemia (ALL) associated with a CSF3R mutation. With the initiation of induction chemotherapy, dasatinib was used in both the induction and consolidation/maintenance phases; the patient was able to achieve a complete hematological response with partial hematologic recovery [40].

Trametinib, a MEK1/2 inhibitor, has shown promising results in in vitro trials when targeting mutated N/KRAS genes, leading to the downregulation of the MAPK pathway and prolonged survival rates in mice transplanted with mutated NRAS genes compared to untreated mice [41]. In phase I/II trials, trametinib showed evidence of achieving a clinical response in myeloid malignancies associated with NRAS mutations, but not including aCML [42]. Furthermore, trametinib was found to play a role in targeting the MAPK signaling pathways in myeloid malignancies associated with wild or truncated CSF3R mutations that fail to activate MEK/extracellular-signal-regulated kinase (ERK) signals [43]. A case report for an 81-year-old male who was treated with trametinib for aCML revealed sustained stabilization of his blood counts over 14 months of therapy [11].

To the best of our knowledge, this is the second published case report of a patient who received trametinib as a targeted therapy for aCML where a sustained response was achieved. Though our patient received trametinib for only four months, she was able to achieve stable blood counts throughout the entire therapy duration.

## Conclusions

Because of its low incidence and lack of standard care recommendations, the diagnosis and treatment of aCML are underdeveloped. To date, the only therapy that has shown some benefits in terms of survival benefits for aCML is HSCT, which is associated with a high rate of relapse due to late diagnosis and the aggressive nature of the disease. With new advancements in NGS, physicians are leaning more toward targeted therapy, which is currently under investigation, mainly in case series or small clinical trials.
